# Akt isoforms in vascular disease

**DOI:** 10.1016/j.vph.2015.03.003

**Published:** 2015-08

**Authors:** Haixiang Yu, Trevor Littlewood, Martin Bennett

**Affiliations:** a Division of Cardiovascular Medicine, University of Cambridge, Box 110, Addenbrooke's Centre for Clinical Investigation, Addenbrooke's Hospital, Hills Road, Cambridge CB2 0QQ, UK; b Department of Biochemistry, University of Cambridge, 80 Tennis Court Road, Cambridge CB2 1GA, UK

**Keywords:** Akt, oncogene identified in the Akt8 retrovirus, ApoE, apolipoprotein E, FOXO, forkhead class O transcription factor, HDL, high-density lipoprotein, OHT, 4-hydroxytamoxifen, IGF, insulin-like growth factor, LDL, low-density lipoprotein, SM22α, smooth muscle protein 22α (*tagln*), TUNEL, terminal deoxynucleotidyl transferase UTP end labelling, VSMC, vascular smooth muscle cell, MMP, matrix metalloproteinase, TIMP, tissue inhibitors of metalloproteinase, ADAM, a disintegrin and metalloproteinase, ECM, extracellular matrix, GSK, glycogen synthase kinase, AAA, abdominal aortic aneurysm, TAA, thoracic aortic aneurysm, eNOS, endothelial nitric oxide synthase, mTOR, mammalian target of rapamycin, Vascular diseases, Atherosclerosis, Akt, Aneurysm

## Abstract

The mammalian serine/threonine Akt kinases comprise three closely related isoforms: Akt1, Akt2 and Akt3. Akt activation has been implicated in both normal and disease processes, including in development and metabolism, as well as cancer and cardiovascular disease. Although Akt signalling has been identified as a promising therapeutic target in cancer, its role in cardiovascular disease is less clear. Importantly, accumulating evidence suggests that the three Akt isoforms exhibit distinct tissue expression profiles, localise to different subcellular compartments, and have unique modes of activation. Consistent with in vitro findings, genetic studies in mice show distinct effects of individual Akt isoforms on the pathophysiology of cardiovascular disease. This review summarises recent studies of individual Akt isoforms in atherosclerosis, vascular remodelling and aneurysm formation, to provide a comprehensive overview of Akt function in vascular disease.

## 1 Introduction

Identified in 1991 [1], [2], the serine/threonine protein kinase Akt (Protein Kinase B, PKB) has emerged as a key mediator of cell proliferation, migration, apoptosis, angiogenesis and metabolism [3], [4], [5], [6], acting downstream of the insulin-like growth factor 1 (IGF1)/phosphatidylinositol 3-kinase (PI3K) signal transduction pathways.

Akt kinases comprise three mammalian isoforms (Akt1, Akt2 and Akt3 or PKBα/β/γ), which are similar in structure but distinct in function. All Akt isoforms contain an N-terminal regulatory pleckstrin homology (PH) domain, a central kinase domain with serine/threonine specificity, and a C-terminal hydrophobic domain [1], [7], [8]. Studies over the last decade using various transgenic and knockout mice have shown that Akt isoforms have partly redundant but also distinct functions in physiological and pathological processes, in part due to different tissue-specific expression of Akt isoforms. For example, while Akt1 is ubiquitously expressed, Akt2 is highly expressed in insulin-responsive tissues such as adipose tissue, liver and skeletal muscle, and Akt3 is highly expressed in the brain [9], [10], [11]. Mice lacking Akt1 are viable but have significant neonatal mortality and are smaller in size than littermate controls. Akt1-null cells display higher rates of apoptosis, indicating a critical role for Akt1 in cell survival [12], [13]. Total body Akt2 knockout mice develop severe type-2 diabetes [13], and cells deficient in Akt2 have impaired glucose utilisation [14]. Akt3-deficient mice display no growth retardation but a 20% smaller brain size, suggesting that Akt3 is important for postnatal brain development [15]. Double knockout mice of Akt isoforms have been generated to assess overlapping roles in homeostasis and development. Mice deficient in Akt1/2 die shortly after birth [15], and Akt1/3 double knockout mice are embryonic lethal [15]. In contrast, Akt2/3 double knockout mice are viable but display impaired glucose homeostasis and growth retardation [9], whereas Akt1^+/−^/Akt2^−/−^/Akt3^−/−^ mice are viable. This indicates that a single allele of Akt1 is sufficient to rescue mice during embryonic development and postnatal survival.

Together with the double and triple knockout studies, there is increasing evidence to show that each Akt isoform possesses non-overlapping functions. In addition to differences in tissue expression, different Akt isoforms localise to distinct subcellular compartments. For example, Akt1 is predominantly localised to the cytoplasm and activated at the plasma membrane, whereas Akt2 is preferentially localised to the cytosol but colocalised with mitochondria. In contrast, Akt3 is localised both to the nucleus and nuclear membranes [16], [17]. However, subcellular localisation also varies between tissues. For example, Akt1 is mainly located in the nucleus in murine peritoneal macrophages and human THP1 cells, whereas Akt3 is located in the cytoplasm. In contrast, Akt1 is predominantly cytosolic and Akt3 is nuclear in hepatocytes [18]. These findings strongly suggest that plasma membrane activation processes may not be required by all Akt isoforms, or by any one isoform in different tissues. A further level of complexity exists based on acute vs chronic activation of Akt. For example, acute Akt1 activation can preserve the contractility of cardiomyocytes whereas chronic activation leads to dilatation and hypertrophy [19]. Therefore, the functions of Akt isoforms are tissue-specific, temporally-regulated and cell context-dependent. Elucidation of the complex role of individual Akt isoforms is therefore required for each tissue and cell type.

## 2 Role of Akt in vascular disease

Significant advances have been made in the last two decades to elucidate the roles of Akt isoforms in cancer, development, metabolism and cardiovascular disease. Several excellent reviews have summarised the molecular regulation of Akt and its downstream signalling [11], [20], [21]. The important role of Akt isoforms in cardiomyocyte survival and cardiac hypertrophy, both under physiological or pathological conditions, has also been reviewed [19], [22], [23], [24], [25]. Here we will summarise the consequences of Akt activation, focusing on cell survival, cell proliferation, and migration, and the role of individual isoforms in cells whose abnormal function underlies vascular disease, including endothelial cells (EC), vascular smooth muscle cells (VSMCs) and macrophages. We will also review recent studies determining the role of individual Akt isoforms in vascular disease, including atherosclerosis, vascular remodelling and aneurysm formation.

### 2.1 Akt in survival signalling

**Fig. 1:**
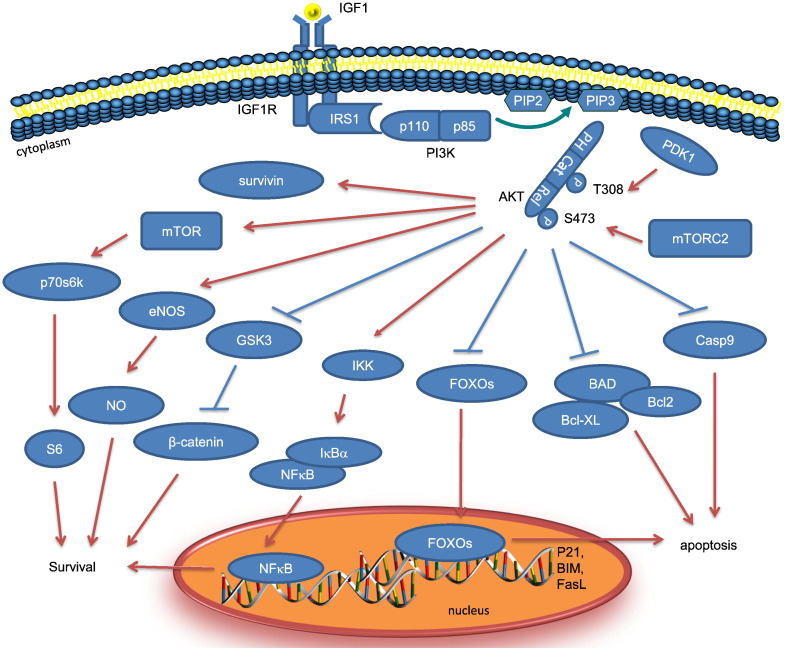
Akt-mediated survival signalling. IGF1R possesses intrinsic tyrosine kinase activity. Once activated by binding to ligands such as insulin/IGF1 to its extracellular domain, receptor autophosphorylation stimulates recruitment, phosphorylation and activation of the adaptor protein IRS1 and which in turn activates PI3K. PI3K activation leads to phosphorylation of PIP2 to PIP3. Plasma membrane-anchored PIP3 and PIP2 attract and bind to the PH domains of Akt and PDK1. Inactive Akt is stimulated via direct binding and phosphorylation of T308 in the central catalytic domain by PDK1 and subsequent phosphorylation of S473 in the C-terminal regulatory domain by mTORC2. Fully activated Akt disassociates from the membrane and further phosphorylates multiple downstream effectors to promote cell survival, either through phosphorylation to activate pro-survival signalling pathways such as NF-kB or to inactivate pro-apoptotic targets such as Bad, Caspase 9, FOXOs and GSK3.

Apoptotic cell death is a common feature in many physiological and pathological processes in the cardiovascular system, including vessel remodelling, atherosclerosis and vascular injury. However, survival signalling pathways, especially the pleiotropic effects of Akt signalling in cardiovascular disease, remain unclear. Critical survival signalling pathways in the cardiovascular system whose effects are mediated through Akt include circulating factors such as insulin-like growth factor-1 (IGF-1) and platelet-derived growth factor (PDGF), cell–cell and cell–extracellular matrix contact, and mechanical signals such as stretch and shear stress from blood flow. Of these, one of the most potent anti-apoptotic growth factors is IGF-1 [26], [27], [28]. The IGF-1 receptor possesses intrinsic tyrosine kinase activity and a number of downstream mediators have been identified, including Insulin Receptor Substrate-1 (IRS-1), PI3-K, and mitogen-activated protein kinase (MAPK). The canonical PI3-K comprises a regulatory p85 subunit and a catalytic p110 subunit that directly phosphorylates the ribosomal protein kinase p70^s6k^ [29], the rho family polypeptide Rac [30], the serum and glucocorticoid-induced kinases SGK [31], [32], [33], and the serine/threonine kinase Akt [34], [35] (Fig. 1). Of all these pathways, much of the anti-apoptotic function of IGF-1 resides in Akt [36], [37]. PI3K activation leads to phosphorylation of phosphoinositides (PtdIns), including PtdIns(3,4,5)P3 and PtdIns(3,4)P2 (PIP3 and PIP2). Plasma membrane-anchored PtdIns(3,4,5)P3 and PtdIns(3,4)P2 bind to the PH domains of Akt and the phosphoinositide-dependent kinase-1 (PDK1). Activated PDK1 stimulates Akt activity by direct binding and phosphorylation of Threonine 308 in the central catalytic domain [38]. Further activation of Akt is regulated through phosphorylation by the mammalian target of rapamycin complex 2 (mTORC2) on Serine 473 in the C-terminal regulatory domain. Once activated by phosphorylation at both sites, Akt disassociates from the membrane and further phosphorylates multiple effectors to activate pro-survival or inactivate pro-apoptotic signalling pathways.

Akt critically regulates apoptosis in many cell types, following a variety of stimuli, and constitutively active (CA) and dominant negative (DN) forms of Akt can inhibit or promote apoptosis respectively [39], [40]. Akt inhibits apoptosis via a variety of mechanisms (Fig. 1), including phosphorylation of the pro-apoptotic proteins Bad and caspase 9 [41], [42]. Bad is a member of the Bcl-2 family, and promotes apoptosis by interacting with other Bcl-2 family member proteins. Phosphorylation of Bad sequesters this protein in an inactive form preventing this association [41]. In contrast, caspase 9 is a cysteine protease intimately involved with regulating and executing apoptosis. Caspase 9 cleavage occurs during formation of a pro-apoptotic complex, the apoptosome, that is responsible for apoptotic signalling in response to the release of cytochrome C from mitochondria. Akt phosphorylation of caspase 9 prevents its cleavage and activation, thus inhibiting apoptosis [42]. Akt also phosphorylates glycogen synthase kinase 3β (GSK3β), which not only regulates glucose metabolism, but also induces apoptosis [43]. Akt can also phosphorylate the O subclass of the forkhead family of transcription factors [44], [45], [46], including FOXO1, FOXO3a, FOXO4 and FOXO6 in humans. Akt phosphorylation of FOXO3a leads to its association with 14-3-3 proteins and retention in the cytoplasm. Survival factor withdrawal leads to FOXO3a dephosphorylation, nuclear translocation, and activation of pro-apoptotic target genes, such as FasL, BIM, PUMA and TRAIL [45]. In addition to inactivation of pro-apoptotic proteins, Akt also upregulates anti-apoptotic genes such as Bcl-2, Bcl-XL [47], and survivin [48]. Akt also activates IκB kinase-α (IKK-α), causing the degradation of IκB, which in turn activates NF-κB [49], and can also increase the transactivation potential of the RelA/p65 subunit of NF-κB [50]. In endothelial cells (EC), Akt1 activates endothelial nitric oxide synthase (eNOS), which promotes cell survival by nitrosylating the reactive cysteine residue in caspases [51], [52]. Although all of these pathways downstream of Akt activation have been described (Fig. 1), not all are activated or important in different cell types. For example, Akt1 prevents apoptosis in vascular smooth muscle cells (VSMCs) predominantly via the inhibition of both FOXO3a and GSK3 [53].

### 2.2 Akt in cell proliferation

VSMC proliferation plays an important role in atherosclerosis, vascular remodelling and neointima formation. However, the role of Akt activation in vascular cell proliferation is unclear, in part because of different effects on normal cells vs. those seen in disease. For example, although Akt is frequently observed to be activated during proliferation and invasion in cancer cells, Akt1 phosphorylation (Ser473) is reduced in VSMCs in human atherosclerotic plaques [53], and reduced phospho-Akt2 levels and phospho-AKT2/total AKT ratios are seen in the media of human aortic aneurysms [54].

Akt1 may be necessary but not sufficient to promote VSMC proliferation. For example, inhibition of Akt signalling with a dominant negative Akt mutant (AA-Akt) potently inhibits VSMC proliferation, DNA synthesis and G1/S exit, associated with increased p21 expression [55]. VSMCs from Akt1-deficient mice also show impaired increase in cell number in culture, although this study did not examine proliferation vs. apoptosis [56]. In contrast, serum and insulin/IGF-1 both stimulate Akt phosphorylation in vitro, but only serum not insulin/IGF-1 promotes MAPK activation and VSMC proliferation. Furthermore, tissue specific activation of Akt1 in endothelial cells suppresses lesion formation after carotid ligation via increased NO production, preservation of a functional endothelial layer, and suppression of VSMC proliferation [57]. Similarly, forced expression of Akt1 in VSMCs does not induce VSMC proliferation in normal arteries, after carotid ligation, or in atherosclerosis [58]. In addition, cell proliferation was 50% lower in VSMCs derived from Akt2/LDLr double knockout mice than LDLr KO mice, suggesting a role of Akt2 in VSMC proliferation, although Akt1 is the major isoform expressed in VSMCs [59].

### 2.3 Akt in cell migration

VSMC migration from the media to the intima occurs in both neointima formation and vessel remodelling. Intimal VSMCs play a protective role in plaque stabilisation, although migrated synthetic VSMCs can also secrete MMPs to degrade ECM, promoting features of plaque vulnerability. Akt potently drives cell motility through dynamic polymerisation and stabilisation of intracellular filaments [60], [61]. Indeed, expression of an activated Akt in fibroblasts was sufficient to remodel actin filaments to promote migration, whereas disruption of Akt activity reversed the migratory effect [62]. Although Akt1 and Akt2 are differentially required for migration by ECs, VSMCs and macrophages, the general effect for Akt activation is to promote cell migration. However, Akt regulates cell migration through distinct effectors and multiple signalling pathways.

Akt is a necessary and sufficient mediator of VEGF-induced EC migration, through eNOS phosphorylation-mediated NO release, phospholipase C-γ (PLC-γ) activation and F-actin reorganisation [63]. Importantly, Akt1, the major isoform regulating NO release in ECs, is also critical for vascular permeability and leukocyte migration into inflamed tissues, evident by reduced neutrophil and monocyte infiltration in Akt1^−/−^ but not in Akt2^−/−^ mice [64]. Akt mediates VSMC migration, which is mainly regulated through MMP activation and ECM degradation. For example, Akt1 mediates VSMC migration through MMP2 secretion, Rac1-GTP activation, and dorsal ruffle formation [56]. Genetic deletion or siRNA knockdown of Akt1 in VSMCs drastically reduces MMP2 expression and VSMC migration, after either serum or PDGF [56], and the reduced migration of Akt1-null VSMCs can be rescued by Akt1 reintroduction. In contrast, how Akt2 promotes VSMC migration is less clear. Although migration of VSMCs from Akt2/LDLR double knockout mice was 50% lower than control LDLR null mice, Akt2 null VSMCs showed increased MMP expression, especially MMP2 and MMP9 [59]. VSMCs of Akt2-null mice showed increased MMP9 but reduced TIMP1 expression. In vitro Akt2 also stimulates TIMP-1 and inhibits MMP9 In human aortic VSMCs through the inhibition of FOXO1 [54].

Migration of macrophages is tightly regulated through chemotactic signalling downstream of Akt2 but not Akt1. Thus, adhesion, spreading and chemokine-induced migration are similar in monocytes from Akt1^−/−^ and control mice [56]. In contrast, migration of Akt2^−/−^ macrophages is impaired when stimulated with monocyte chemoattractant protein-1 (MCP-1/CCL2) or macrophage colony-stimulating factor (M-CSF or CSF-1) [65]. Akt2^−/−^ macrophages have reduced C-C chemokine receptor type 2 (CCR2) expression, blunted Rac-1 activity and disrupted F-actin structures, in comparison with Akt1-null or wt macrophages [66]. Moreover, Akt2 also plays a dominant role in promoting neutrophil migration [67].

## 3 Role of different Akt isoforms in cell types comprising vascular lesions

### 3.1 Akt isoforms in endothelial cells

Endothelial cells (ECs) predominantly express Akt1 [68], [69] which mediates EC survival and migration [24], [70], with important roles in processes such as angiogenesis and regulation of vascular tone [71]. Various stimuli such as vascular endothelial growth factor (VEGF) activate Akt in ECs, resulting in Akt-dependent phosphorylation of endothelial nitric oxide synthase (eNOS) to promote NO release; NO is a critical regulator of vascular tone and blood flow, and also regulates vascular remodelling and angiogenesis. The isoform-substrate specificity for Akt1 vs. Akt2 in EC has been examined recently using conditional knockout mouse models of Akt1^−/−^ and Akt2^−/−^ in combination with phosphoproteomic analysis, demonstrating that eNOS is a preferential target of Akt1[69]. Indeed, loss of Akt1 but not Akt2 in ECs inhibited retinal angiogenesis, indicating a non-redundant function of Akt1 in angiogenesis. Furthermore, Akt1 but not Akt2 in ECs uniquely phosphorylates protein substrates implicated in cardiovascular disease, including FOXOs and eNOS [69].

### 3.2 Akt isoforms in vascular smooth muscle cells

Akt1 is the predominant isoform expressed in VSMCs, where a major role is prevention of apoptosis [6], [54], [56], [58]. For example, Akt1 is both necessary and sufficient for the survival of cultured rat VSMCs following oxidative stress, and dominant negative inhibition of endogenous Akt suppresses IGF1-dependent survival in VSMCs [53], [72], confirming also that Akt is a major downstream target of the IGF1/PI3K signalling pathway. Akt1-null VSMCs show increased susceptibility to serum starvation and stress-induced apoptosis [73], whereas expression of constitutive active Akt1 inhibits oxidative stress-induced VSMC apoptosis both in vitro and in vivo, through negative regulation of FOXO3a and GSK3. In addition, as described above, Akt1 also regulates VSMC proliferation and migration, the latter due to reduced Rac-1 activity and MMP-2 secretion [56].

### 3.3 Akt isoforms in macrophages

The expression and activation of Akt isoforms are more complex in macrophages than in ECs and VSMCs. Akt1 and Akt2 are both highly expressed to similar levels in macrophages [59], [65], [66], whereas Akt3 accounts for only ~ 25% of total Akt expression [18]. Akt regulates macrophage survival, NO synthesis, cytokine secretion and programming [74]. For example, Akt1 promotes macrophage IFN-β expression by negative regulation of GSK3β or direct phosphorylation of β-catenin [75]. Recent studies show that Akt1 and Akt2 differentially regulate macrophage polarisation [76], [77]. Akt1^−/−^ but not Akt2^−/−^ macrophages show hyper-sensitivity to lipopolysaccharide (LPS), and AKT1^−/−^ macrophages display a pro-inflammatory M1 phenotype, whereas Akt2^−/−^ macrophages show an anti-inflammatory M2 phenotype. In vitro, Akt1 deficiency has no effect on macrophage adhesion, migration or lipoprotein uptake, but potently suppresses oxidised low-density lipoprotein (oxLDL)-induced macrophage apoptosis [73]. In contrast, Akt2^−/−^ macrophages have increased expression of M2 markers, reduced foam cell formation and impaired migration [65], [66]. The opposing effects of Akt1 and Akt2 in macrophage M1/M2 polarisation, inflammation and cholesterol accumulation indicate that Akt isoforms might have completely different or even opposing effects on atherosclerosis. Finally, as a minor form of Akt in macrophages, Akt3 is not involved in cell survival, but suppresses foam cell formation by reducing lipoprotein uptake and promoting acetyl-CoA acetyltransferase-1 (ACAT-1) degradation [18].

## 4 Role of Akt in vascular disease

### 4.1 Akt isoforms in atherosclerosis

The atherosclerotic plaque comprises an accumulation of vascular smooth muscle cells, inflammatory cells (macrophages, mast cells) and immune cells (T lymphocytes, dendritic cells) with both intracellular and extracellular lipid and debris. Endothelial dysfunction and macrophage migration are both early and constant features of atherosclerosis, whereas, the dynamic balance of VSMC proliferation, migration, dedifferentiation and death plays an important role in both atherogenesis and plaque stability. For example, VSMC apoptosis directly induces multiple features of plaque vulnerability in atherosclerosis, including fibrous cap thinning, necrotic core expansion, and both local and systemic inflammation [78]. Chronic low-level VSMC apoptosis also promotes atherogenesis, calcification and medial degeneration (cystic medial necrosis), characterised by depletion of VSMCs and ECM degradation [79]. Macrophage apoptosis is also present in advanced plaques, promoting necrotic core formation and release of pro-inflammatory cytokines [80].

Both Akt1 and Akt2 are expressed in blood vessels including the aorta, femoral and carotid arteries. Akt1 is the predominant isoform expressed in endothelial cells and VSMCs [6], [68], whereas Akt2 is mostly expressed in fibroblasts [4]. Akt3 is barely detectable in the vasculature. Atherosclerosis results in changes in expression and phosphorylation of both Akt and its downstream signalling molecules. For example, medial VSMCs maintain Akt activity and FOXO3a phosphorylation, whereas plaque intimal VSMCs show reduced Akt phosphorylation and increased expression of active (unphosphorylated) FOXO3a [53], suggesting that Akt activity and Akt-mediated VSMC survival may play a protective role in atherogenesis.

Indeed, genetic depletion of Akt1 induces endothelial cell dysfunction, reduces VSMC migration and survival, promotes atherosclerosis and coronary artery obstruction, and induces features of plaque vulnerability [56], [73]. The detrimental effect of Akt1 deficiency on atherosclerosis was not reversible by reconstitution with wild-type bone marrow [73], demonstrating that the changes are mediated by vessel wall cells.

Akt1 not only has multiple downstream targets but also can have distinct targets in different cells. For example, Akt1 stimulates eNOS in endothelial cells but not in VSMCs. Chronic Akt activation may even induce apoptosis, by feedback inhibition of PI3K [81]. Studies using whole body Akt1 knockout mice have therefore been supplemented by studies using tissue-specific knockout or overexpression. For example, VSMC-specific expression of a hydroxytamoxifen-activated Akt1 allele (Akt-ER™) reduced VSMC apoptosis, reduced plaque formation, and increased plaque collagen and VSMC content, indicating that VSMC Akt1 protects against atherosclerosis and promotes plaque stability. In addition to well-established FOXO3a targets p27, cyclin D1, bim, bcl6 and gadd45α, the apoptosome component Apaf1 was shown to be a novel downstream apoptosis mediator regulated by Akt/FOXO3a signalling.

In contrast, a deleterious role for Akt2 in atherosclerosis has recently been elucidated. In vitro, loss of Akt2 impairs VSMC proliferation, migration and collagen synthesis and alters the expression of MMPs and TIMPs. However, Akt2 deficiency reduced atherosclerotic plaques in both carotid arteries and aortic roots, characterised by reduced collagen content, enlarged necrotic core and elevated apoptosis [59], despite Akt2^−/−^/LDLR^−/−^ mice having both diabetes and increased cholesterol levels. Although it was suggested that increased VSMC apoptosis in the absence of Ak2 might be responsible for the phenotype, Akt1 is the major isoform expressed in VSMCs, and a more likely effect may be due to loss of macrophage Akt2. (see below).

Macrophage Akt1 and Akt2 also have different effects on atherosclerosis. Transplantation of foetal liver cells from Akt1 or Akt2 deficient mice to LDLR^−/−^ mice showed that Akt2^−/−^ → LDLR^−/−^ but not Akt1^−/−^ → LDLR^−/−^ or WT^−/−^ → LDLR^−/−^ dramatically reduced early atherosclerotic lesion formation, and also suppressed advanced atherosclerotic lesions [65]. Bone marrow transplantation (BMT) studies demonstrated a very similar protective role of macrophage Akt2 deficiency in atherosclerosis progression [66]. In both studies, in vitro data supported a role for Akt2 in macrophage migration, inflammation and polarisation, suggesting that Akt2 deficiency reduces atherosclerosis through impaired macrophage function.

**Fig. 2:**
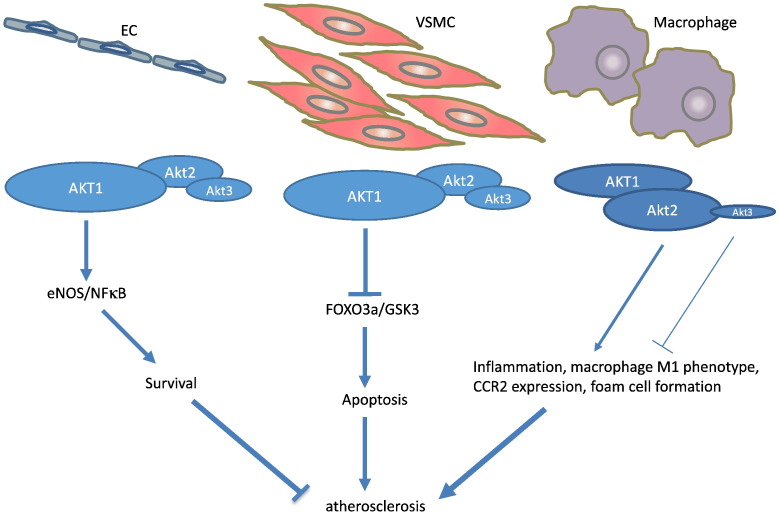
Akt isoforms in EC, VSMC and macrophage function in atherosclerosis. ECs and VSMCs predominantly express Akt1. In ECs, Akt1 activates eNOS and NF-κB to promote cell survival. In VSMCs, Akt1 phosphorylates FOXO3a and GSK3 to inhibit apoptosis. Akt1 plays a protective role in EC and VSMC survival to inhibit atherosclerosis. In macrophages, Akt2 but not Akt1 potently induces inflammation, the M1 phenotype, foam cell formation and CCR2-mediated migration, aggravating atherosclerosis. As a minor isoform in macrophages, Akt3 inhibits macrophage foam cell formation and is found to be anti-atherogenic.

Finally, although predominantly expressed in brain, Akt3 also has a protective role in atherosclerosis [18]. Genetic ablation of Akt3 in ApoE^−/−^ mice led to a 2-fold increase in atherosclerotic lesions, and bone marrow transplantation of Akt3^−/−^/ApoE^−/−^ to ApoE^−/−^ mice also resulted in a significant increase of atherosclerosis [18]. Akt3 deficiency did not affect macrophage apoptosis, but promoted macrophage cholesterol accumulation, lipoprotein uptake and foam cell formation in vitro, via stabilising Acetyl-Coenzyme A acetyltransferase 1 (ACAT1). The findings of transgenic, genetic deletion and tissue-specific deletion studies for individual Akt isoforms in atherosclerosis are summarised in Table 1, and the role of Akt isoforms in different cell types and their effects on atherosclerosis are shown in Fig. 2.

### 4.2 Akt isoforms in vascular remodelling

Vascular remodelling occurs both physiologically and pathologically in response to changes in flow. Thus, flow reduction results in remodelling leading to reduced lumen size and vessel wall thickness, and vice versa. Remodelling is regulated by Akt in both ECs and VSMCs, particularly Akt1. For example, expression of an endothelial cell-specific constitutively active Akt1 transgene significantly attenuated neointima formation in carotid arteries after ligation [57], mediated through inhibition of neointimal cell apoptosis and Akt1-mediated eNOS activation in ECs. Similarly, although Akt1 activation in VSMCs does not promote proliferation in vivo or induce neointimal formation, it potently inhibited medial VSMC apoptosis and negative remodelling after carotid ligation [58]. Consistent with these studies, Akt1-deficient mice show a 2-fold increase in neointima formation compared with Akt1^+/+^ mice, and Akt1 also modulates phenotypic conversion of VSMCs, highlighting a protective role of Akt1 in vascular remodelling [82].

### 4.3 Akt isoforms in aneurysm formation

Aortic aneurysms are characterised by > 1.5 fold increase in diameter of the aorta, and both the thoracic aorta (TAA) or abdominal aorta (AAA) can be affected [83]. The aneurysm is characterised by medial degeneration, loss of VSMCs, VSMC apoptosis, activation of matrix metalloproteinases (MMPs) and inflammation [84], [85], [86].

Akt isoforms are implicated in aneurysm formation, although there is a degree of controversy in the literature. For example, Akt2 appears to have a protective role in aortic aneurysm formation and dissection [54]. Akt2 but not Akt1 levels were significantly reduced in the whole aortic wall of human TAAs, and phospho-Akt^473^ levels and phospho-Akt/total Akt ratios were significantly reduced in TAA versus control tissues, indicating reduced activation of Akt. Although Akt2^−/−^ mice did not develop dissection spontaneously, Angiotensin II challenge led to aortic aneurysms, with significant increase in the diameter of aortic segments, lesions and ruptures in Akt2^−/−^ vs. control mice. Severe elastic fibre disruption was observed followed by increased apoptosis and inflammatory cell infiltration in the vessel wall of Angiotensin II-infused Akt2**^−/−^** mice. Furthermore, expression of MMP9 was increased whereas TIMP1 was decreased in lesion segments in these animals. Further experiments suggest that Akt2 inhibits MMP-9 and stimulates TIMP-1 expression by preventing FOXO1-mediated MMP-9 transcription and GATA1-regulated TIMP-1 transcription [54].

The role of Akt in AAA development was examined using an elastase-treated mouse model [87]. Elastase-perfused aortas showed increased phosphorylation of Akt^308^, but not Akt^473^ in male mice, which correlated with increased AAA formation. Interestingly, this study reported consistently elevated Akt activity both in human and mouse AAA. These differing findings may be due to different animal models and human tissue sites, and also the effect of isoform compensation, where knockout of one isoform may result in compensatory increases in other isoforms. Importantly, treatment with a broad-spectrum Akt inhibitor significantly reduced Akt phosphorylation, but only resulted in a small reduction in AAA development, suggesting that phosphorylation of Akt is involved in AAA initiation, but is not critical for AAA progression.

## 5 Conclusions and future perspectives

Significant progress has been made towards understanding the precise roles of individual Akt isoforms in vascular disease, including atherosclerosis, vascular remodelling and aneurysm formation. Based on findings from tissue-specific transgenic or knockout animal models, Akt1 is the predominant isoform expressed in the vasculature including EC and VSMCs. Akt1 activation potently mediates cell survival and protects against atherogenesis and negative vessel remodelling. Akt3, a minor isoform of total Akt, may also have similar anti-atherogenic actions to Akt1. In contrast, Akt2 is detrimental in atherosclerosis, through aberrant macrophage migration, inflammation, polarisation, lipid uptake, and foam cell formation. Thus, Akt1 and Akt2 are not only differentially expressed in the vasculature but also have distinct and even opposite functions in vascular disease. Bearing in mind the weak expression of Akt2 in ECs and VSMCs, the protective role conferred by Akt2 in aneurysm formation and dissection needs to be further validated.

The current studies also leave many unanswered questions. For example, do the current animal findings correlate with human tissues, and what is the clinical significance of altered Akt activity in human disease? What level of Akt activation leads to distinct and non-compensated actions achieved by individual Akt isoforms? How do we account for the apparent disparate roles of Akt2 in diabetes and atherosclerosis, when atherosclerosis is the major cause of death in diabetes? Are Akt or isoforms of Akt therapeutic targets considering the wide tissue distribution and fundamental role in multiple processes? The last decade has clarified the role of individual isoforms in animal tissues; the next decade should clarify their relevance to human vascular disease.

## Figures and Tables

**Table 1:** Studies and effects of Akt isoforms in atherosclerosis in mice.

	Targeted Akt isoform	Phenotype/morphology	Mechanism	Reference
Akt1 Tg	SM22αAkt1ER/ApoE^−/−^	↓ atherosclerosis,	VSMC survival	[58]
Genetic depletion	Akt1^−/−^/ApoE^−/−^	↑ atherosclerosis, ↑ coronary lesions	Inflammation, EC and macrophage apoptosis	[73]
Akt2^−/−^/LDLR^−/−^	↑ insulin resistance, ↓ atherosclerosis,	VSMC survival, collagen homeostasis	[59]
Akt2^−/−^/LDLR^−/−^	No effect	–	[65], [66]
Akt3^−/−^/ApoE^−/−^	↑ atherosclerosis, ↑ foam cells	Foam cell formation	[18]
BMT	Akt1^−/−^ → ApoE^−/−^	No effect	–	[66]
Akt1^−/−^ → LDLR^−/−^	No effect	–	[65]
Akt2^−/−^ → LDLR^−/−^	↓ atherosclerosis	Macrophage migration and polarisation	[65], [66]
Akt3^−/−^ → ApoE^−/−^	↑ atherosclerosis, ↑ foam cells	Foam cell formation	[18]
